# RRx-001 Increases Erythrocyte Preferential Adhesion to the Tumor Vasculature

**DOI:** 10.3390/ijms22094713

**Published:** 2021-04-29

**Authors:** Vinay P. Jani, Robert Asaro, Bryan Oronsky, Pedro Cabrales

**Affiliations:** 1 Department of Bioengineering, University of California, San Diego, La Jolla, CA 92093, USA; v1jani@eng.ucsd.edu; 2 Department of Structural Engineering, University of California, San Diego, La Jolla, CA 92093, USA; scipio394@gmail.com; 3 EpicentRx Inc., 4445 Eastgate Mall, Suite 200, San Diego, CA 92121, USA; boronsky@epicentrx.com

**Keywords:** RRx-001, tumor microenvironment, RBC adhesion, shear stress

## Abstract

Red blood cells (RBCs) serve a variety of functions beyond mere oxygen transport both in health and pathology. Notably, RRx-001, a minimally toxic pleiotropic anticancer agent with macrophage activating and vascular normalization properties currently in Phase III trials, induces modification to RBCs which could promote vascular adhesion similar to sickle cells. This study assessed whether RBCs exposed to RRx-001 adhere to the tumor microvasculature and whether this adhesion alters tumor viability. We next investigated the biomechanics of RBC adhesion in the context of local inflammatory cytokines after treatment with RRx-001 as a potential mechanism for preferential tumor aggregation. Human HEP-G2 and HT-29 tumor cells were subcutaneously implanted into nu/nu mice and were infused with RRx-001-treated and Technetium-99m (^99m^Tc)-labeled blood. RBC adhesion was quantified in an in vitro human umbilical vein endothelial cell (HUVEC) assay under both normoxic and hypoxic conditions with administration of either lipopolysaccharide (LPS) or Tumor necrosis alpha (TNFα) to mimic the known inflammation in the tumor microenvironment. One hour following administration of ^99m^Tc labeled RBCs treated with 10 mg/kg RRx-001, we observed an approximate 2.0-fold and 1.5-fold increase in ^99m^Tc-labeled RBCs compared to vehicle control in HEPG2 and HT-29 tumor models, respectively. Furthermore, we observed an approximate 40% and 36% decrease in HEP-G2 and HT-29 tumor weight, respectively, following treatment with RRx-001. To quantify RBC adhesive potential, we determined $\tau_{50}$, or the shear stress required for 50% disassociation of RBCs from HUVECs. After administration of TNF-α under normoxia, $\tau_{50}$ was determined to be 4.5 dynes/cm^2^ (95% CI: 4.3–4.7 dynes/cm^2^) for RBCs treated with 10 μM RRx-001, which was significantly different (*p* < 0.05) from $\tau_{50}$ in the absence of treatment. Under hypoxic conditions, the difference of $\tau_{50}$ with (4.8 dynes/cm^2^; 95% CI: 4.6–5.1 dynes/cm^2^) and without (2.6 dynes/cm^2^; 95% CI: 2.4–2.8 dynes/cm^2^) 10 μM RRx-001 treatment was exacerbated (*p* = 0.05). In conclusion, we demonstrated that RBCs treated with RRx-001 preferentially aggregate in HEP-G2 and HT-29 tumors, likely due to interactions between RRx-001 and cysteine residues within RBCs. Furthermore, RRx-001 treated RBCs demonstrated increased adhesive potential to endothelial cells upon introduction of TNF-α and hypoxia suggesting that RRx-001 may induce preferential adhesion in the tumor but not in other tissues with endothelial dysfunction due to conditions prevalent in older cancer patients such as heart disease or diabetic vasculopathy.

## 1. Introduction

RRx-001 is a minimally toxic pleiotropic anticancer agent with macrophage activating, CD47 downregulating and vascular normalizing properties currently under investigation in phase III clinical trials [1,2]. A therapeutic on its own, RRx-001 is also a chemo- and radiosensitizer, augmenting traditional radiation and chemotherapeutics for solid tumor therapy primarily in the absence of systemic toxicities since the compound has not been associated with any dose limiting side effects in over 300 patients treated [3,4]. Furthermore, the compound is a protective agent against chemotherapy and radiation-induced cytotoxicity in normal tissues, further highlighting its clinically utility. These seemingly counterintuitive effects of RRx-001 have been attributed to induction of nuclear factor erythroid 2–related factor 2 (Nrf2) in normal tissues, which is cytoprotective, while simultaneously inducing cytotoxicity in the tumor environment through downregulation of the anti-apoptotic Bcl2, downstream of Nrf2, as well as through CD47 antagonism, tumor associated macrophage (TAM) polarization, and vascular normalization [4]. The diverse functions and mechanisms surrounding RRx-001 cytotoxicity make it an attractive target for research. For instance, RRx-001 has been evaluated in melanoma [5], glial tissue (malignant glioblastoma) [5,6], lung epithelium (small cell lung carcinoma) [2,7], ovarian epithelium [4], and more recently, erythrocytes [1]. Interestingly, much like in sickle anemia, RRx-001 induces a dose dependent translocation of phospholipid phosphatidylserine (PS) and increased hemoglobin (Hb) oxidation in RBCs [8]. However, the mechanisms surrounding these effects remain underexplored.

Sickle cell red blood cells (sRBCs) are known to accumulate preferentially in the tumor vasculature, with the highest abundance observed at the interface between the tumor and normal tissue [9]. This phenomenon is likely due to the complex function of the tumor microenvironment, characterized by pockets of hypoxia, acidic conditions, reduced blood flow, and ischemia, all of which promote sickling and subsequently, sRBC aggregation [10]. The mechanism of sickling is well described and involves precipitation of sickle cell hemoglobin (Hb) within the microvasculature followed by subsequent formation of long Hb polymers, which further distort the biconcave erythrocyte structure, impair deformability, and increase PS expression [11]. This clinically manifests as vaso-occlusive crises, which continue to be a problem for clinical management of sickle cell disease [12]. However, under normal physiologic conditions, sickling is an uncommon phenomenon in the absence of external stressors (e.g., infection induced acute chest syndrome) as sickling time is less than normal RBC capillary transit time. Importantly, the acidic and hypoxic tumor microenvironment, along with increased transit time secondary to increase vascular tortuosity and decreased blood flow provide sufficient stress to induce sickling, as shown by blood oxygen dependent imaging (BOLD) [9]. These data all demonstrate that the unique conditions and stressors imposed by the tumor microenvironment provide the necessary conditions for sickling and thus RBC aggregation.

As described, RRx-001 can induce increased externalization of PS, as is observed in sickle cell disease, which is thought to be due to increased Hb oxidation [8,13]. The mechanism of RRx-001 induced Hb oxidation involves increased nitric oxide production with subsequent (1) covalent binding of RRx-001 to the cysteine residue on the beta chain of hemoglobin, which under hypoxic conditions, increases conversion of nitrate to nitric oxide, and (2) RRx-001 dependent reaction with glutathione (GSH) and other thiols, which releases nitric oxide and other NO_x_ variants [14,15,16]. When RRx-001 binds to Hb or reacts with GSH, nitro groups are lost, resulting in direct production of NO variants, all of which may induce NO mediated pathways. RRx-001 depletion of GSH and other antioxidant cysteine residues further contribute to oxidative stress. Oxidative injury can also result from formation of RRx-001-GSH adduct intermediaries, which further depletes GSH. Under hypoxic conditions, RRx-001 functionalized Hb has also been observed to produce more nitric oxide under hypoxic conditions. These mechanisms explain RRx-001’s ability to increase denaturation and precipitation of hemoglobin. Thus, RRx-001 mediates oxidative injury in erythrocytes by increased production of nitric oxide and NO derived compounds, which result in increased reactive oxygen species and increased translocation of phospholipid phosphatidylserine in RBCs. The tumor microenvironment can also upregulate expression of the phospholipid phosphatidylserine receptor from hypoxia, increased inflammatory cytokines, and increased free heme. Together these data imply that RRx-001-modified RBCs may interact in unique ways with the tumor, which can be exploited for therapeutic potential [17].

This study exploits the observation that RRx-001 increases RBC membrane PS, while the tumor microvascular endothelium increases phospholipid phosphatidylserine receptor (PSR) to explore the effects of RRx-001 on RBCs within the tumor microenvironment. To this end, we assessed whether RBCs exposed to RRx-001 adhere to the tumor microvasculature and whether this adhesion alters tumor viability. We next investigated the biomechanics of RBC adhesion in the context of local inflammatory cytokines after treatment with RRx-001 as a potential mechanism for preferential tumor aggregation. We observed that RRx-001 treated RBCs preferentially aggregate in two known human cell line derived tumor models. Furthermore, we observed that RRx-001 treated RBCs demonstrated increased adhesive potential to endothelial cells upon introduction of TNF-α and hypoxia, both of which increase endothelial PSR expression, suggesting that that the compound may induce preferential adhesion in the tumor.

## 2. Results

### 2.1. RRx-001 Localizes to RBCs

**Figure 1:**
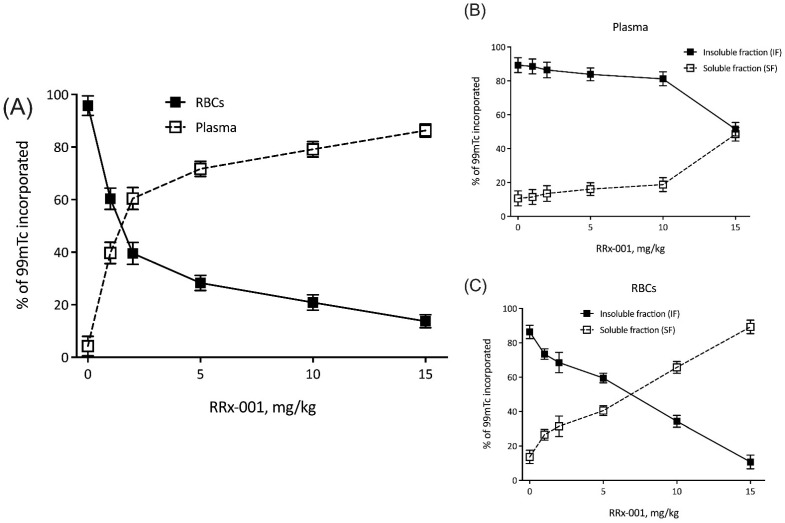
RRx-001 localizes to RBCs. Shown here are percent of ^99m^Tc incorporated into (**A**) plasma and RBCs, (**B**) insoluble and soluble fractions of plasma, and (**C**) insoluble and soluble fractions of RBCs, all as a function of RRx-001 dose (mg/kg).

To test whether RRx-001 localized to RBCs and preferentially associated with hemoglobin in vitro, we treated RBCs with increasing concentrations of RRx-001, which is known to associate with Hb by oxidation of mercapto residues [14], and ^99m^Tc, which is known to bind to mercapto residues at the Hb beta subunit [18]. These results are summarized in Figure 1A. Briefly, as expected, increasing concentrations of RRx-001 resulted in a dose dependent decrease in RBC incorporated ^99m^Tc, with 14 ± 3% ^99m^Tc incorporated in RBCs at a dose of 15 mg/kg RRx-001. To determine whether RRx-001 predominantly associated with Hb, radioactivity of the soluble and insoluble fractions of both plasma (Figure 1B) and RBCs (Figure 1C) were probed, with the insoluble fraction of RBCs assumed to predominantly consist of Hb. In plasma, there was no change in the relative % ^99m^Tc incorporated into insoluble and soluble protein fractions of plasma between 0 and 10 mg/kg RRx-001. At a dose of 15 mg/kg RRx-001, 52 ± 4% ^99m^Tc was observed in the insoluble fraction, suggesting no preference of ^99m^Tc labeling for either protein fraction at this concentration. However, in RBCs, there was an RRx-001 dose dependent decrease in % ^99m^Tc incorporated in the insoluble protein fraction, with 11 ± 4% ^99m^Tc incorporated in the insoluble fraction, suggesting that RRx-001 displaced radiolabeled ^99m^Tc by associating with Hb.

### 2.2. RRx-001 Increases RBC Adhesion to the Endothelium

**Figure 2:**
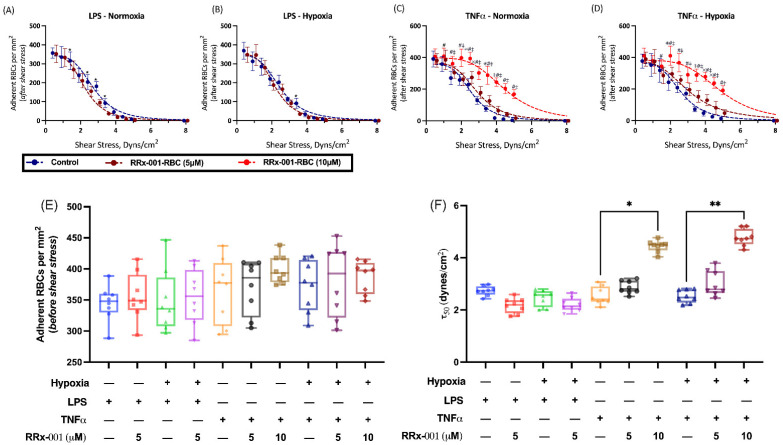
RRx-001 Induces Increased RBC Adhesion to the Endothelium. Adherent RBCs per mm^2^ versus Shear Stress were fit to an inverse sigmodal curve and are shown for untreated and RRx-001 treated cells at 5 and 10 mg/kg under (**A**) LPS-induced inflammation and Normoxia, (**B**) LPS-induced inflammation and hypoxia, (**C**) TNFα induced inflammation and normoxia, (**D**) TNFα induced inflammation and hypoxia (* *p* < 0.05 Control vs. RRx-001 5 μM, # *p* < 0.05 Control vs. RRx-001 10 μM, ‡ *p* < 0.05 RRx-001 5 μM vs. RRx-001 10 μM). Fit parameters for inverse sigmoidal curves, namely (**E**) Adherent RBCs per mm^2^ (before application of shear stress), and (**F**) $\tau_{50}$, or the minimum shear stress for 50% disassociation of RBCs, are shown for the same parameters (* *p* < 0.03, ** *p* < 0.002, for Holm-Sidak multiple comparisons).

**Figure 3:**
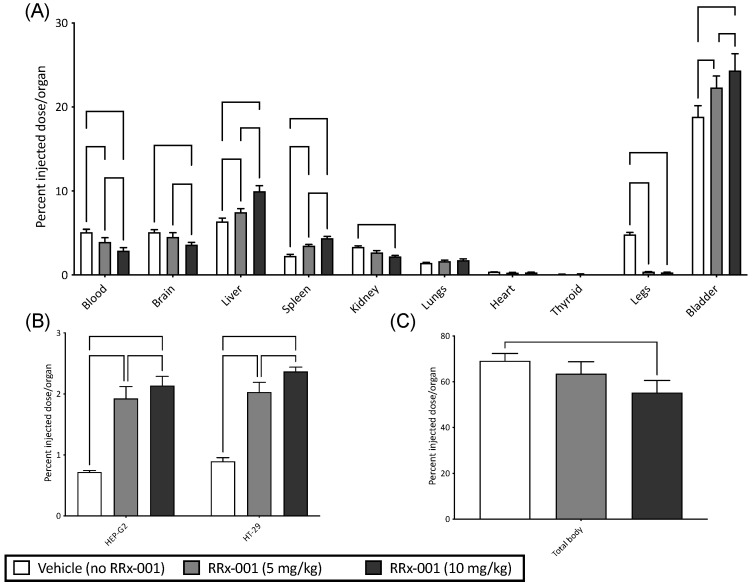
RRx-001 Treated RBCs Preferentially Localize to Solid Tumors. Shown here are percent injected dose/organ of ^99m^Tc labeled blood for (**A**) blood, brain, liver, spleen, kidney, lungs, heart, thyroid, legs, and bladder (**B**) HEP-G2 and HT-29, and (**C**) total body ^99m^Tc, for vehicle control (no RRx-001) and RRx-001 at concentrations of 5 and 10 mg/kg. (for Holm-Sidak multiple comparisons (**B**) or Dunn’s multiple comparisons (**C**)).

Given that RRx-001 treated RBCs preferentially localized to tumors in our animal models, we next sought to better understand the mechanism of this phenomenon. We hypothesized that RRx-001 increased RBC adhesive potential to the tumor endothelium. To characterize RBC adhesive potential after treatment of RRx-001, we utilized an in vitro HUVEC assay under hypoxic conditions with administration of either LPS or TNFα to mimic the hypoxia and cytokine mediated inflammation known to characterize the tumor microenvironment and induce endothelial phosphatidylserine receptor expression [17,19,20]. These results are summarized in Figure 2. To quantify RBC adhesion, adherent RBCs per mm^2^ was plotted as a function of applied shear stress and fit to an inverse sigmoidal curve, and $\tau_{50}$, or the shear stress required for 50% disassociation of RBCs from HUVECs, was calculated. Under LPS induced inflammation and normoxia, RRx-001 did not significantly change $\tau_{50}$, 3.0 dynes/cm^2^ and 2.6 dynes/cm^2^ before and after 5 μM RRx-001 treatment, respectively (Figure 2A,F). This did not appreciably change under hypoxic conditions (Figure 2B,F). However, under TNFα induced inflammation, treatment with 10 μM RRx-001 induced a significant rightward shift in the curve (Figure 2C,D), with $\tau_{50}$ being significantly elevated (*p* < 0.05) after RRx-001 treatment, 3.1 dynes/cm^2^ and 4.4 dynes/cm^2^ before and after RRx-001 treatment, respectively (Figure 2F). Similar results were observed under hypoxic conditions (Figure 2D,F; *p* < 0.05). Importantly, no significant differences were observed in the number of adherent RBCs before application of shear stress (Figure 3E). Together, these results suggest that RRx-001 increased RBC adhesive potential to the endothelium under TNFα induced inflammation and hypoxia, as in the tumor microenvironment.

### 2.3. RRx-001 Increases RBC Membrane Phospholipid Phosphatidylserine Expression

To further investigate the mechanism of increased RBC adhesion to the tumor microvascular endothelium, we measured the expression of several membrane receptors known to be affected by RRx-001, namely CD 36, CD 71, PS, and CD 47 (Table 1). We observed a significant increase in expression of all receptors (CD 36, CD 71, PS, and CD 47) measured. Specifically, there was a 6.8-fold increase in PS expression (*p* = 0.002) on the erythrocyte membrane. In the context of previous data showing an increase in PS receptor expression in the tumor microvascular endothelium [17], these data suggest that an increase in the PS and PS-receptor interaction after RBC treatment with RRx-001 is a viable mechanism for the observed increase in RBC adhesion.

### 2.4. RRx-001 Treated RBCs Preferentially Localize to Solid Tumors and Decrease Tumor Viability

To test whether RRx-001 treated RBCs preferentially localized to tumor cells, we utilized two experimental tumor models of human HEP-G2 (hepatocellular carcinoma) and HT-29 (colorectal carcinoma) cells implanted in nu/nu mice to account for the T cell tumor response. Both models were infused with RRx-001 treated- and ^99m^Tc labeled-blood; results are summarized in Figure 3. The organ distribution of RRx-001 and vehicle in all experiments is summarized in Figure 3A. Briefly, there was a significant increase in ^99m^Tc labeled blood with and without RRx-001 observed in the bladder and liver compared to other organs. These results, while unsurprising, suggest that most RRx-001 modified RBCs were observed to undergo hepatic elimination and some renal clearance (Figure 3A), presumably modified RBCs were cleared in the liver, while hemoglobin in the kidneys, which was consistent with previous reports of RRx-001 pharmacodynamics [21]. In both tumor models, we observed a dose dependent and significant increase (*p* < 0.05 compared to vehicle) in %RRx-001 injected in both tumor models as assessed by fraction of ^99m^Tc radiolabeled RBCs (Figure 3B). Total body radioactivity (Figure 3C) was observed to decrease with increasing dose of RRx-001 (*p* < 0.05 at 10 mg/kg compared to vehicle) likely as we are underestimating the volume of treated RBCs in the tumor due to competition between ^99m^Tc and RRx-001 Hb binding. Together these results suggest that RRx-001 treated RBCs preferentially localize to tumors in our experimental models.

**Figure 4:**
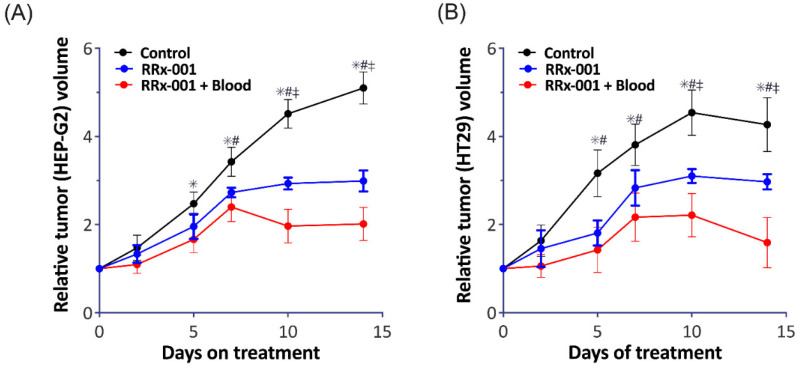
RRx-001 Treated RBCs Augment Tumor Cytotoxicity Compared with RRx-001 treatment alone. Shown here is the relative tumor volume in both our (**A**) HEP-G2 and (**B**) HT29 tumor models after treatment with vehicle (black), RRx-001 alone (blue), and RRx-001 treated RBCs, denoted here as RRx-001 + Blood (red). We observed a significant reduction in both tumor models after treatment with both RRx-001 and RR-001 + blood compared to vehicle control. After 10 days, RRx-001 + blood have an even greater reduction in tumor volume compared with RRx-001 alone. (* *p* < 0.05 Control vs. RRx-001, # *p* < 0.05 Control vs. RRx-001 + Blood, ‡ *p* < 0.05 RRx-001 vs. RRx-001 + Blood).

We next investigated tumor viability after treatment with RRx-001. First, we observed an approximate 40% and 36% decrease in HEP-G2 and HT-29 tumor weight, respectively, following administration of RRx-001 treated RBCs. We then measured tumor viability over time with treatment of RRx-001 alone and RRx-001 treated RBCs (Figure 4) in both HEP-G2 and HT-29 tumor models. Specifically, in HEP-G2 cells, there was a significant reduction in tumor volume after treatment with RRx-001 treated blood and RRx-001 alone compared with control 7 days post treatment. At 10- and 15-days post treatment, the group treated with RRx-001 treated blood showed a significant reduction in tumor volume compared to treatment with RRx-001 alone. A similar trend was observed in the HT29 tumor model. In summary, we observed that RRx-001 treated RBCs resulted in a significant reduction (*p* < 0.05) in tumor weight in both models compared to vehicle control and treatment with RRx-001 alone after 10 days. Importantly, treatment with RRx-001 alone resulted in a significant reduction in tumor volume (*p* < 0.05) compared to vehicle control, but it appears that RBCs treated with RRx-001 augmented tumor cytotoxicity, consistent with what we previously reported [8]. Together, these results further demonstrated that RRx-001 treated RBCs are more cytotoxic to tumor cells compared with RRx-001 alone.

## 3. Discussion

The principal findings of this study are: (1) RRx-001 localizes to RBCs likely by associating with cysteine residues on Hb, (2) RRx-001 treated RBCs preferentially localize to tumors by increasing RBC adhesion to the tumor endothelium in our experimental tumor models, likely by increasing phospholipid phosphatidylserine expression, and (3) localization of RRx-001 treated RBCs affects tumor viability. Importantly, this study demonstrates that RRx-001 treatment of RBCs produces sufficient membrane PS expression to increase RBC adhesion in cases when endothelial PSR expression is increased (e.g., after treatment with TNFα and hypoxia). Furthermore, based on this proposed mechanism, an RRx-001 dependent increase in RBC adhesion is independent of tumor cell lineage, as observed in our study.

We observed that ^99m^Tc incorporation in the insoluble fraction of RBCs decreased with increased RRx-001 dose, suggesting that RRx-001 displaced radiolabeled ^99m^Tc, which is known to bind to cysteine residues of Hb. These results suggest that RRx-001 preferentially interacts with the insoluble protein fraction, namely Hb, of RBCs, and interacts with Hb cysteine residues further demonstrating the distinct affinity RRx-001 has for RBCs. ^99m^Tc is known to interact preferentially with thiol containing residues (e.g., cysteine) on the beta chain of hemoglobin [18,22]. RRx-001 is known to interact with similar cysteine residues, and in fact, is known to specifically bind to the cysteine 93 residues on the beta chain of hemoglobin with exquisite specificity [14]. Whether cysteine 93 binding is the predominant mechanism of ^99m^Tc displacement in this assay is unknown; RRx-001 may in fact bind other cysteine residues in hemoglobin, further displacing ^99m^Tc. Importantly, the association between RRx-001 and cysteine 93 residues accounts for only 10–30% of RRx-001-human Hb interactions, suggesting that interactions with GHS and other thiolate containing groups within the erythrocyte may in fact account for the majority of the drug’s oxidative effects in RBCs [14]. Subsequent NO production during hypoxia may be both from RRx-001’s interactions with cysteine 93 residues and other antioxidants (e.g., GSH) within the erythrocyte [14,16]. The observed preferential localization of RRx-001 to RBCs in our study suggests that oxidation and depletion of the intra-erythrocyte GSH pool may be the predominant mechanism of RRx-001 mediated cytotoxicity. Furthermore, depletion of GSH by RRx-001 severely limits the erythrocyte’s ability to reduce reactive oxygen species (ROS) formation, further increasing the probability of Hb oxidation [14].

The results from our study further support the hypothesis that RBCs are the critical effector of cytotoxicity after RRx-001 treatment. As discussed above and demonstrated by several studies [8,14], RRx-001 mediated cytotoxicity and chemo sensitization depends on increased RBC oxidation and NO production. Several mechanisms for NO production exist, though most relevant here are NO production from βcys93 residue interactions and interactions with and depletion of GSH (and other antioxidant species) [8,14]. Important to our study, however, is the idea that increased Hb oxidation results in translocation of PS on the outer membrane of the RBC. Such a translocation has several implications, including increased RBC aggregation, discussed in detail below, and decreased tumor viability by means of occlusion of the hypoxic tumor vasculature [8]. This mechanism is further supported by our observation that RRx-001 directly increases PS expression on the erythrocyte membrane. Vascular occlusion also induces a subsequent vascular normalization from shunting and redirection of blood flow through more efficient vessels, leading to the chemo-sensitization and radio-sensitization commonly observed with RRx-001 treatment. RBCs are then endocytosed by the tumor endothelium, resulting in the release of iron and free heme, which increases oxidative stress. Free heme as well as RRx-001 mediated downregulation of CD47 expression on tumor cells and SIRPα expression on macrophages induces a shift from the low phagocytic M1 phenotype to the high phagocytic M2 phenotype in tumor cells [8,23]. Thus, RRx-001 mediated RBC PS externalization may be of pivotal importance to the function of RRx-001 as a chemosensitizer and as a therapeutic for cancer treatment.

Our results show that RRx-001 increases RBC adhesion to the endothelium under TNFα induced inflammation and hypoxia, which explain the observed preferential localization to tumor cells. We hypothesize that the mechanism of increased adhesion is a consequence of an RRx-001 mediated increase in erythrocyte membrane PS and increased interaction with PSR on the endothelium. To induce PSR expression on HUVECs, we utilized TNFα and LPS, both of which are known to stimulate endothelial PSR expression [17]. These effects likely underestimate the true effects RRx-001 on RBC adhesion in vivo, as free heme release from RRx-001 treated RBCs can upregulate PSR after interaction with macrophages, which were not included in our assay. Interestingly, we observed a differential response in changes in RBC adhesive potential with TNFα and LPS, namely that RRx-001 significantly increased adhesion only with TNFα stimulation. These results, are, however, not unexpected, as TNFα is a more potent stimulator of PSR expression in the endothelium compared to LPS, though we admit that the differences in RRx-001 dose may also explain these results [17]. Importantly, treatment of endothelium with TNFα and LPS requires activation of PS expression on RBCs to increase adhesion, further suggestion that our results are explained by an increase in PS-PSR interactions following RRx-001 treatment [17]. The requisite presence of TNFα, which is presumably more abundant in tumor vs. non-tumor endothelium perhaps explains the absence of RRx-001-related side effects in treated vasculopathic patients with cancer. Future studies should aim to further elucidate these effects.

### Limitations

Our study has several limitations. ^99m^Tc can bind to imidazole rings in histidine residues, which are abundant in Hb [22]. As a result, ^9m^Tc radiolabeling in general may underestimate the extent to which RRx-001 binds and interacts with thiol containing residues in Hb, though this is a limitation of the assay. Furthermore, the competition between radiolabeled ^99m^Tc and RRx-001 for cysteine residues on Hb results in an underestimation of the number of RBCs present within the tumor vasculature, as there are fewer ^99m^Tc labeled RBCs with increasing RRx-001 dose. We also did not directly measure proteins directly indicative of tumor cell death. Given that several apoptotic pathways exist, future studies should measure these proteins to better understand the mechanism by which RRx-001 induces tumor cell death. In our adhesion studies, we did not directly determine measure PSR expression, though we did measure PS expression post RRx-001 treatment. Our results remain speculative as to whether increased PS-PSR interactions are a viable mechanism for the observed increase in RBC adhesion. Future studies should aim to further investigate the specific mechanism of RRx-001 induced increase of PS expression on erythrocyte membranes and further elucidate downstream effects.

## 4. Materials and Methods

### 4.1. RRx-001 RBC Treatment

Fresh blood was collected from C57BL/6J mice, weighing 20–24 g into syringes containing Anticoagulant Citrate Dextrose (ACD). For all animals in this study, the NIH Guide for the Care and Use of Laboratory Animals was followed. The study protocol was approved by the local animal care committee. Briefly, plasma and erythrocyte isolation were achieved via centrifugation (2000 rpm, 5 min), and the buffy coat was discarded. Plasma and packed RBCs were fixed. RRx-001 was mixed with blood to achieve concentrations equivalent to dosing mice with 1, 2, 5, 10, and 15 mg/kg. Blood mixtures were incubated with Tin (II) chloride (SnCl_2_) at 1 mg/mL for 10 min. After incubation, 1 mCi 99mTc was added to this mixture, and the mixture was then incubated for 10 min. The sample mixture was then centrifuged (2500 rpm, 5 min), and the plasma and packed RBCs were separated. Aliquots of the plasma (P) and RBCs were precipitated with trichloroacetic acid (5%) and were then centrifuged (1500 rpm, 5 min) to isolate the soluble (SF) and insoluble (IF) fractions of the plasma and RBCs. Radioactivity of P, RBCs, SF-plasma, IF-plasma, SF-RBCs, and IF-RBCs were determined in a well counter, and the percentage of ^99m^Tc incorporated (% ^99m^Tc) was calculated.

### 4.2. Red Cell Preparation

Blood was collected from healthy volunteers into heparinized syringes and transferred into small tubes with CPD for a final anticoagulant to a blood ratio of approximately 1:7. Blood was centrifuged, and the buffy coat was discarded. RBCs were used fresh within 24 h of collection.

### 4.3. Human Endothelial Culture and Activation

Human umbilical vein endothelial cells (HUVECs) were purchased from the American Type Culture Collection (Manassas, VA abbrev, USA, Umbilical Vein Endothelial Cells; Normal). For adhesion experiments, HUVECs were cultured in gelatin-coated sterile glass coverslips and cultured at 37 °C at 5% CO_2_ until confluent in Vascular Cell Basal media supplemented with 10% fetal calf serum (FCS) (Gibco, Life Technologies, Waltham, MA, USA), and 1% (100 μg/mL) penicillin/(100 μg/mL) streptomycin (Gibco, Life Technologies). Prior to the RBC adhesion experiment, HUVECs monolayers were incubated with endotoxin (Lipopolysaccharides from *Escherichia coli* O111:B4 at 200 ng/mL; Sigma Aldrich, St Louis, MO, USA) or TNF-α (10 ng/mL) in culture media at 37 °C for 8 h.

### 4.4. RBC Adhesion Study

RBC adhesion to activated HUVECs was determined under continuous laminar flow of untreated RBCs (RBCs) or RBCs incubated with RRx-001 at (5 µM) (RRx-001-RBCs). Cells were diluted to a 2% hematocrit in 5% human serum albumin (Albuminar, Armour Pharmaceutical, Kanakee, IL, USA) solution in PBS. Confluent HUVEC layers were incubated with cells for 30 min before perfusion. After incubation with RBCs, suspended cells were washed three times with PBS, then the monolayers were perfused at incremental shear stresses with RBC free media for 2 min. The number of adhered RBCs was quantified before and after each shear exposure in 10 randomly selected sites within 2 min under no shear conditions. Studies were performed under oxygenated and deoxygenated conditions. For hypoxic conditions, HUVECs cells were incubated at 37 °C in a modular chamber flushed with 1% O_2_, 5% CO_2_ and 94% N_2_, and red cells suspension and the solutions used in the adhesion study were also deoxygenated with 100% N_2_ solution.

### 4.5. Erythrocyte Receptor Expression after RRx-001 Treatment

Treated and untreated red blood cells suspension (200 μL, about 2 × 10^7^ total RBCs) were incubated for 60 min at room temperature with 100 μL diluted anti-CD36 monoclonal antibody (EPR6573, ab133625), anti-CD71 monoclonal antibody (EPR20584, ab214039), or anti-CD47 monoclonal antibody (EPR21794, ab218810) at a concentration of 5 μg/mL (all antibodies from ABCAM, Cambridge, MA, USA). These preparations then were washed twice in PBS and incubated with secondary fluorescent antibody. In addition, another sample was incubated with Annexin V-FITC Apoptosis Staining at 10 μg/mL (ab273273, ABCAM). Positive quantification was completed by hemocytometer.

### 4.6. Tissues and Solid Tumor RBC Accumulation Experimental Models

A total of 24 6-week-old athymic mice (nu/nu) were entered into the study and implanted with 20 µL (1 × 10^6^ cells) of tumor suspension (either HEP-G2 cells or HT-29 cells). For all animals in this study, the NIH Guide for the Care and Use of Laboratory Animals was followed. The study protocol was approved by the local animal care committee. Briefly the tumor suspension consisted of human HEP-G2 cells (liver carcinoma cells) and HT-29 cells (colorectal adenocarcinoma cells), which were suspended in Dulbecco’s Modified Eagle Medium (DMEM) at 1 × 10^5^ cells/µL for subcutaneous implantation. Two weeks after implantation of the tumor suspension, blood from nu/nu mice was collected and treated with RRx-001, and then labeled with ^99m^Tc. Two concentrations of RRx-001, 5 mg/kg and 10 mg/kg along with a vehicle control were used, with n = 4/tumor type/group. This mixture was administered to the mice. Mice were euthanized 1 h after administration. After euthanasia, the vital organs, and both tumors were quickly isolated, weighed, and counted. The percentage of dose to weight was then determined.

### 4.7. Red Cell Dependent Activity of RRx-001 in Solid Tumors

In a different set of mice (nu/nu) either HEP-G2 cells (n = 4) or HT-29 (n = 4) cells were implanted as described before. One week after tumor implantation, blood from nu/nu mice was collected and treated with RRx-001 (5 mg/kg) and infused via tail vein. In addition, two control groups were used for the study, in one, animals received RRx-001 (5 mg/kg) in PBS without RBCs, and the other was an untreated control. RRx-001 treatments were repeated every 72 h. Tumor size was measured over 2 weeks, and animal were euthanized at the end of the study.

### 4.8. Statistical Analysis

Results are presented as mean ± standard deviation. All statistical calculations and graphics were performed and generated with a commercially available software package (GraphPad Prism 9.1, San Diego, CA, USA). To test differences in tumor localization, a 2-way ANOVA with post hoc Holm Sidak multiple comparisons between vehicle and RRx-001 doses was used. All data assessing RBC adhesion were fit to a three-parameter inverse sigmoidal curve. Differences in total body ^99m^Tc and fit parameters were assessed using a nonparametric Kruskal-Wallis test with post hoc Dunn’s multiple comparisons. For all tests, *p* < 0.05 was accepted as statistically significant.

## 5. Conclusions

In summary, we showed that RBCs treated with RRx-001 preferentially adhere in tumors, likely due to increased RBC membrane PS expression secondary to an increase in Hb oxidation. RRx-001 treated RBCs demonstrated increased adhesive potential to endothelial cells upon introduction of TNF-α and hypoxia, suggesting that the process is indeed PS-PSR mediated. Future studies should aim to further elucidate the mechanisms by which PS expression is increased in RRx-001 and whether other mechanisms of an RRx-001 mediated increase in RBC adhesion to the tumor endothelium exist.

## Figures and Tables

**Table 1:** Changes in Erythrocyte Receptor Expression after RRx-001 Treatment. * *p* < 0.05 relative to controls. # *p* < 0.05 (paired *t*-test) relative to pre-treatment group.

Membrane Receptor	Controls Mean ± SD	RRx-001		*p*-Value
		Before Mean ± SD	After Mean ± SD	
CD 36 (%)	0.5 ± 0.4	0.6 ± 0.3	2.5 ± 1.0 *^#^	0.013
CD 71 (%)	0.9 ± 0.4	1.0 ± 0.8	4.2 ± 1.2 *^#^	0.003
PS (%)	0.8 ± 0.4	1.2 ± 0.6	9.4 ± 1.4 *^#^	0.002
CD 47 (%)	0.8 ± 0.3	0.9 ± 0.4	1.9 ± 0.8 *^#^	0.042

## Data Availability

The raw data supporting the conclusions of this article will be made available by the authors, without undue reservation, to any qualified researcher.
